# Supplementary material for: UBAP2L contributes to formation of P-bodies and modulates their association with stress granules
Source: J Cell Biol. 2024 Jul 15;223(10):e202307146. doi: 10.1083/jcb.202307146 (PMC11248227; doi:10.1083/jcb.202307146)
Supplement: Table S1 — shows cell lines used in this study and their sources. [file JCB_202307146_TableS1.docx]

**Table S1.** Cell lines used in this study and their sources.

| **Cell line** | **Source** |
| --- | --- |
| U2OS-WT | ATCC |
| U2OS-WT Tet Repressor (U2OS-tr) | (Kedersha et al., 2016) |
| U2OS-tr G3BP1/G3BP2 KO double (G3BP1/2 KO) | (Kedersha et al., 2016) |
| U2OS-tr FXR1/FXR2/FMR1 3KO (FXR1/FXR2/FMR1 KO) | (Sanders et al., 2020; Smith et al., 2020) |
| U2OS UBAP2L KO (UBAP2L KO) | (Sanders et al., 2020) |
| GFP-UBAP2L/UBAP2L KO (c3) | This paper |
| U2OS t/o-APEX-G3BP1-WT/G3BP1/2 KO | (Marmor-Kollet et al., 2020) |
| U2OS t/o-GFP-UBAP2L/UBAP2L KO (c8) | This paper |
| U2OS t/o-GFP-LALA/UBAP2L KO (c17) | This paper |
| U2OS t/o-GFP-UBAP2L/G3BP1/2 KO (c2) | This paper |
| U2OS t/o-GFP-UBAP2L in FXR1/FXR2/FMR1 KO – drug-selected pool | This paper |
| U2OS t/o-GFP-FXR1 in FXR1/FXR2/FMR1 KO (c13) | This paper |
