# Supplementary material for: UBAP2L contributes to formation of P-bodies and modulates their association with stress granules
Source: J Cell Biol. 2024 Jul 15;223(10):e202307146. doi: 10.1083/jcb.202307146 (PMC11248227; doi:10.1083/jcb.202307146)
Supplement: Table S2 — shows information for antibodies used in this study. [file JCB_202307146_TableS2.docx]

**Table S2.** Information for antibodies used in study.

| **Antigen** | **Host species** | **Catalog number** | **Source** | **IF dilution** | **WB dilution** |
| --- | --- | --- | --- | --- | --- |
| 4-ET | Goat | sc-13455 | Santa Cruz Biotechnology, Inc. | 1:200 | N/A |
| Ago2 | Mouse | 014-22023 | Wako | 1:200 | N/A |
| DAZAP1 | Rabbit | A303-984A | Bethyl Labs | 1:200 | N/A |
| DCP1A | Mouse | Sc-100706 | Santa Cruz Biotechnology, Inc. | 1:500 | N/A |
| DDX6 | Rabbit | A300-461A | Bethyl Labs | 1:500 | N/A |
| EDC3 | Mouse | Sc-365024 | Santa Cruz Biotechnology, Inc. | 1:500 | N/A |
| eIF3b  note: antibody discontinued, therefore used Invitrogen eIF3b as well. | Goat | sc-16377 | Santa Cruz Biotechnology, Inc. | 1:500 | N/A |
| eIF3b | Rabbit | PA5-117928 | Invitrogen | 1:500 | NA |
| eIF4E | Mouse | sc-9976 | Santa Cruz Biotechnology, Inc. | 1:200 | N/A |
| eIF4G | Rabbit | SC-11373 | Santa Cruz Biotechnology, Inc. | 1:500 | N/A |
| FXR1 | Goat | sc-10554 | Santa Cruz Biotechnology, Inc. | 1:200 | N/A |
| G3BP1 | Mouse | sc-365338 | Santa Cruz Biotechnology, Inc. | 1:500 | 1:1000 |
| HEDLS/  P70 S6 kinase* | Mouse | sc-8418 | Santa Cruz Biotechnology, Inc. | 1:200 | N/A |
| PABP | Rabbit | 10970 | Protein Tech | 1:200 | N/A |
| TIA-1 | Goat | sc-1751 | Santa Cruz Biotechnology, Inc. | 1:500 | N/A |
| UBAP2 | Rabbit | A304-626A | Bethyl Labs | 1:500 | 1:1000 |
| UBAP2L | Rabbit | A300-534A | Bethyl Labs | 1:200 | 1:1000 |
| XRN1 | Rabbit | A300-443A | Bethyl Labs | 1:200 | N/A |
| YB1 | Goat | sc-18057 | Santa Cruz Biotechnology, Inc. | 1:500 | N/A |
| YBX3 (ZONAB) | Rabbit | A300-070A | Bethyl Labs | 1:200 | N/A |
| Cy3 AffiniPure^TM^ Goat Anti-Rabbit IgG (H + L) |  | 111-165-003 | Jackson Immuno | 1:2000 |  |
| Cy3 AffiniPure^TM^ Goat Anti-Mouse IgG (H + L) |  | 711-165-140 | Jackson Immuno | 1:2000 |  |
| Cy3 AffiniPure^TM^ Donkey Anti-Goat IgG (H + L) |  | 705-165-003 | Jackson ImmunoResearch | 1:200 |  |
| Cy5 AffiniPure^TM^ Donkey Anti-Goat IgG (H + L) |  | 705-175-147 | Jackson ImmunoResearch | 1:200 |  |
| Cy5 AffiniPure^TM^ Goat Anti-Rabbit IgG (H + L) |  | 711-175-152 | Jackson ImmunoResearch | 1:200 |  |
| Cy^TM^5 AffiniPure Goat Anti-Mouse IgG (H + L) |  | 115-005-003 | Jackson ImmunoResearch | 1:200 |  |
| Cy^TM^2 AffiniPure Goat Anti-Rabbit IgG (H + L) |  | 111-005-003 | Jackson ImmunoResearch | 1:200 |  |
| Cy^TM^2 AffiniPure Donkey Anti-Goat IgG (H + L) |  | 705-005-003 | Jackson ImmunoResearch | 1:200 |  |
| Cy^TM^2 AffiniPure Donkey Anti-Goat IgG (H + L) |  | 705-225-147 | Jackson ImmunoResearch | 1:200 |  |
| Peroxidase-Conjugated AffiniPure Donkey anti-Mouse | Donkey | 715-035-150 | Jackson ImmunoResearch | NA | 1:5000 |
| Peroxidase-Conjugated AffiniPure Donkey anti-Goat) at 1:5000 | Donkey | 711-005-152 | Jackson ImmunoResearch | NA | 1:5000 |

* Indicates cross-reactivity with HEDLS (Kedersha and Anderson, 2007) and thus used to detect HEDLS.
