# Supplementary material for: UBAP2L contributes to formation of P-bodies and modulates their association with stress granules
Source: J Cell Biol. 2024 Jul 15;223(10):e202307146. doi: 10.1083/jcb.202307146 (PMC11248227; doi:10.1083/jcb.202307146)
Supplement: Table S3 — shows details of siRNAs used in this study. [file JCB_202307146_TableS3.docx]

**Table S3.** Details of siRNAs used in this study.

| **Target** | **siRNA** | **Source** |
| --- | --- | --- |
| UBAP2 (Human) Entrez Gene 55833 | siGENOME Human UBAP2 siRNA – SMARTpool, targeting: GAAUUCUGCUAGUCCAGUA  GCACAACCAGUACCUCGUA  CCAGUGGACUACUAUGGAA  GAGCCUAGCUAAUAAUCCA | Horizon Discovery, cat no.  M-013168-00 |
| UBAP2L (Human) Entrez Gene 9898 | siGENOME Human UBAP2L (9898) siRNA – SMARTpool, targeting:  GCAGAUAUCUCAGGGCUAA  GCUAAAGGCGGCAGUACUA  CCUGGGAGAUGGUCGGGAA  CAACACAGCAGCACGUUAU | Horizon Discovery, cat no. M-021220-01 |
| Non-Targeting control | siGENOME Non-Targeting siRNA Pool #2, targeting:  UAAGGCUAUGAAGAGAUAC  AUGUAUUGGCCUGUAUUAG  AUGAACGUGAAUUGCUCAA  UGGUUUACAUGUCGACUAA | Horizon Discovery, cat no. D-001206-14-05 |
| GFP | forward sequence 5′-GGCTACGTCCAGGAGCG-3′ | IDT Technology, custom synthesis |
