# Supplementary material for: UBAP2L contributes to formation of P-bodies and modulates their association with stress granules
Source: J Cell Biol. 2024 Jul 15;223(10):e202307146. doi: 10.1083/jcb.202307146 (PMC11248227; doi:10.1083/jcb.202307146)
Supplement: Table S4 — shows plasmids used to generate t/o cell lines or for transient transfection in this study. [file JCB_202307146_TableS4.docx]

**Table S4.** Plasmids used to generate t/o cell lines or for transient transfection in this study.

| **Plasmid** | **Source** |
| --- | --- |
| pcDNA4 t/o-GFP-UBAP2L | (Youn et al., 2018) |
| pcDNA4 t/o-GFP-LALA | (Youn et al., 2018) |
| FM5-GFP-UBAP2L dRGG (1-123; 205-1087) | Gift from David Sanders |
| FM5-GFP-UBAP2L dDUF (1-494; 528-1087) | Gift from David Sanders |
| FM5-GFP-UBAP2L Full-length (FL; 1-1087) | (Sanders et al., 2020) |
| FM5-GFP-UBAP2L 495-527 (DUF) | Gift from David Sanders |
| FM5-GFP-UBAP2L 124-204 (contains RGG) | Gift from David Sanders |
| FM5-GFP-UBAP2L 91-1087 | (Sanders et al., 2020) |
| FM5-GFP-UBAP2L 205-1087 | Gift from David Sanders |
| FM5-GFP-UBAP2L 291-1087 | (Sanders et al., 2020) |
| FM5-GFP-UBAP2L 495-1087 | Gift from David Sanders |
| FM5-GFP-UBAP2L 528-1087 | (Sanders et al., 2020) |
