# Supplementary figures and images for: UBAP2L contributes to formation of P-bodies and modulates their association with stress granules
Source: J Cell Biol. 2024 Jul 15;223(10):e202307146. doi: 10.1083/jcb.202307146 (PMC11248227; doi:10.1083/jcb.202307146)

merged cholor. & chemi.

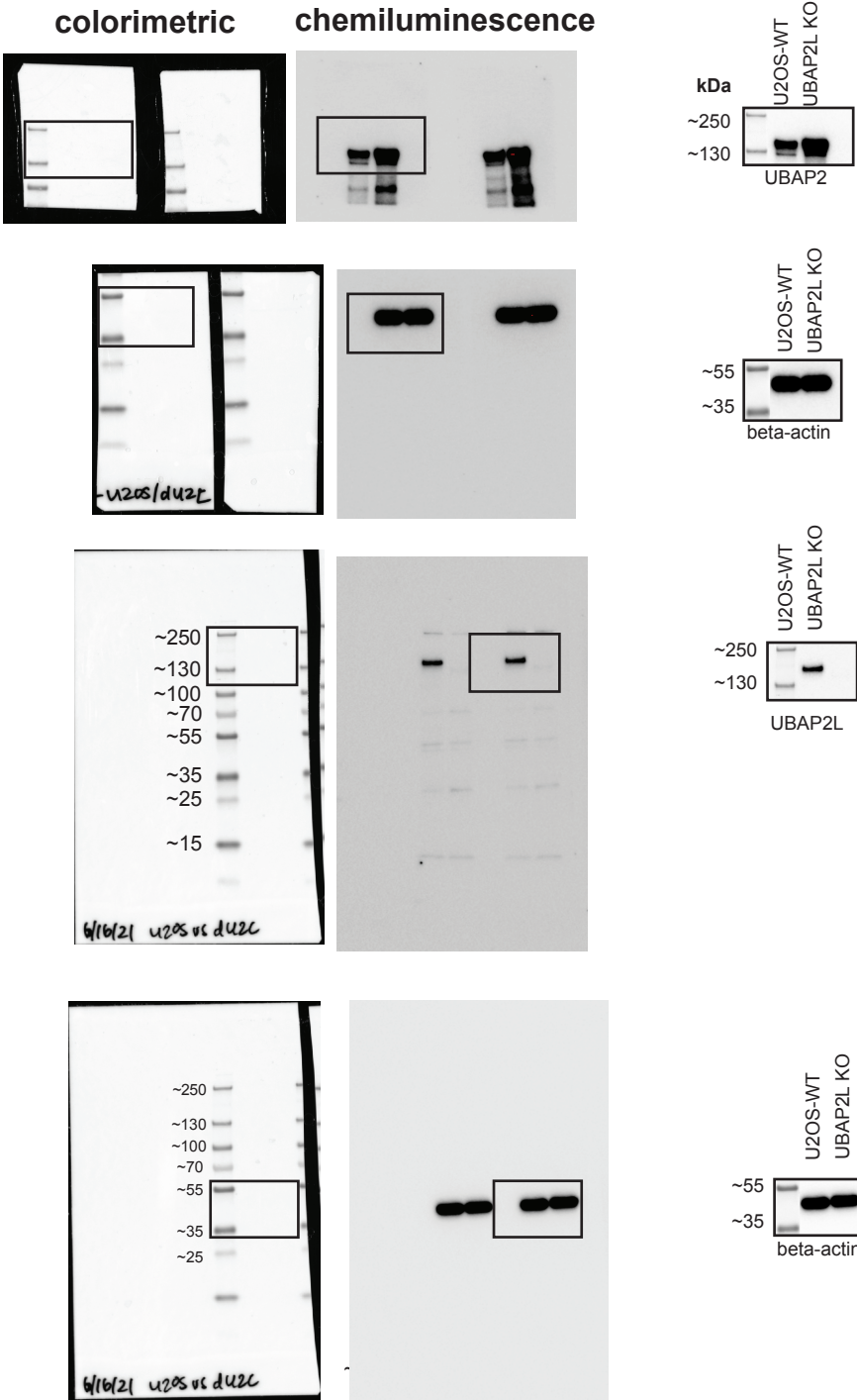

Supplement: SourceData F1 — is the source file for Fig. 1. [file JCB_202307146_SourceDataF1.pdf]

Colorimetric

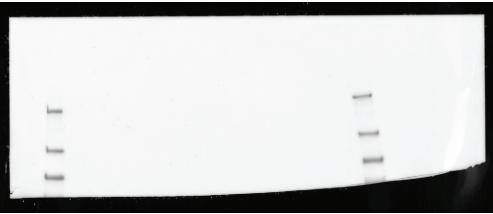

Chemiluminescence

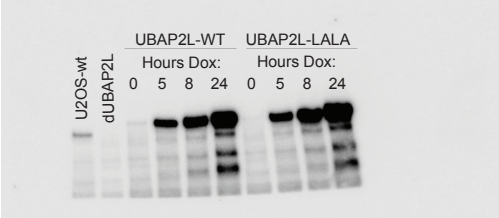

merged color. & chemi.

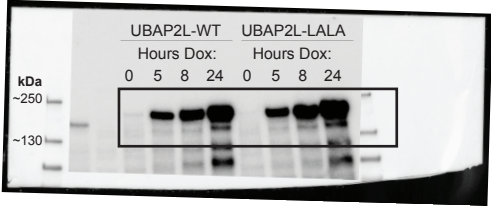

UBAP2L

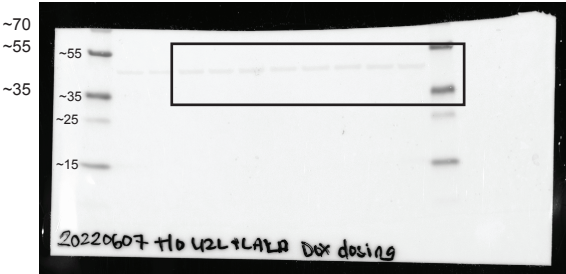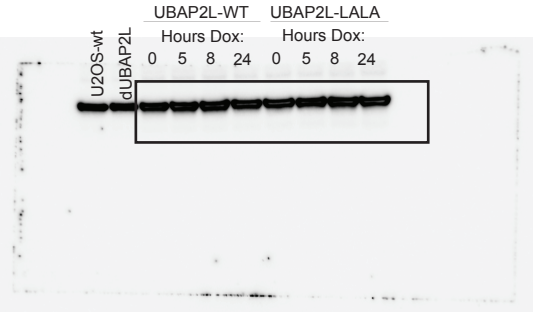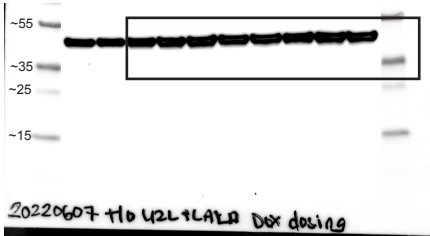

beta actin

Supplement: SourceData F4 — is the source file for Fig. 4. [file JCB_202307146_SourceDataF4.pdf]

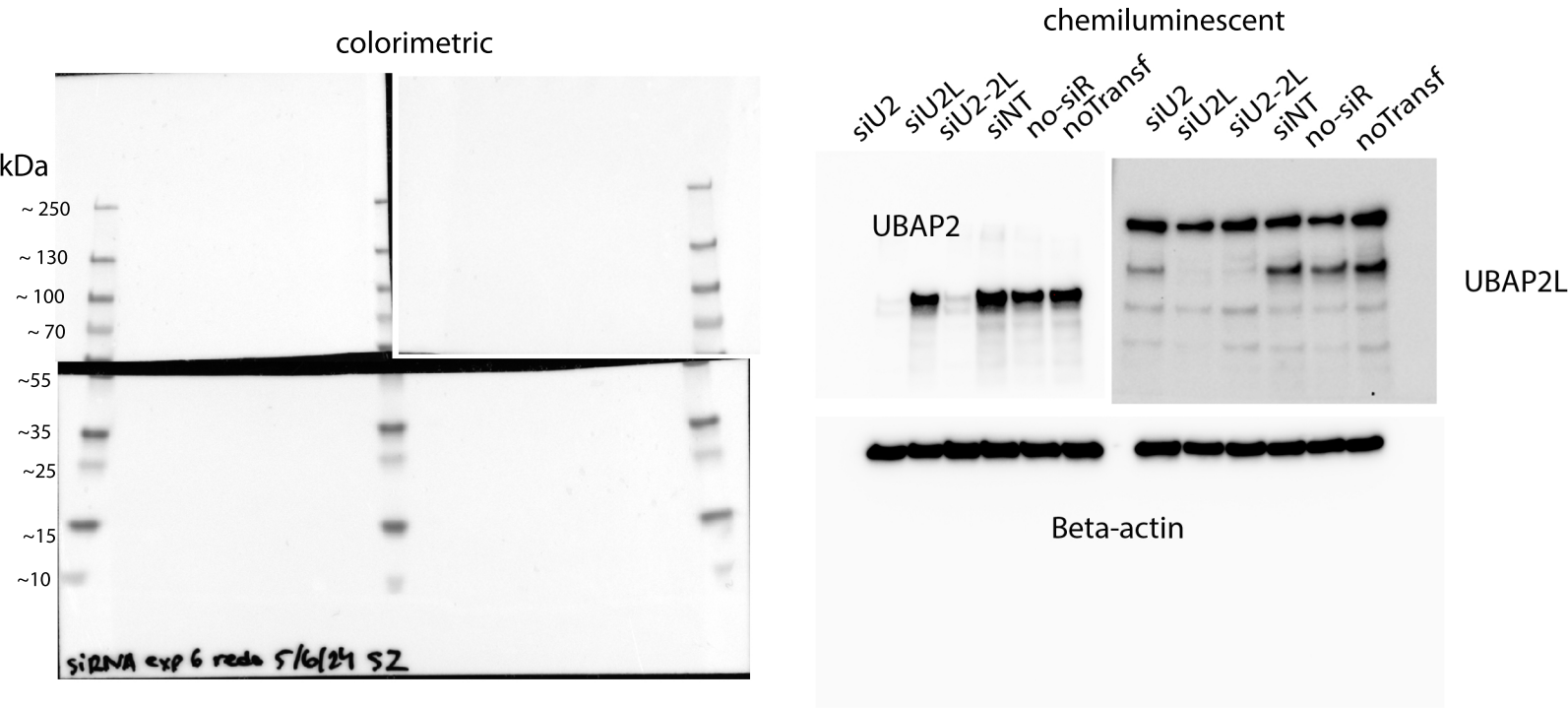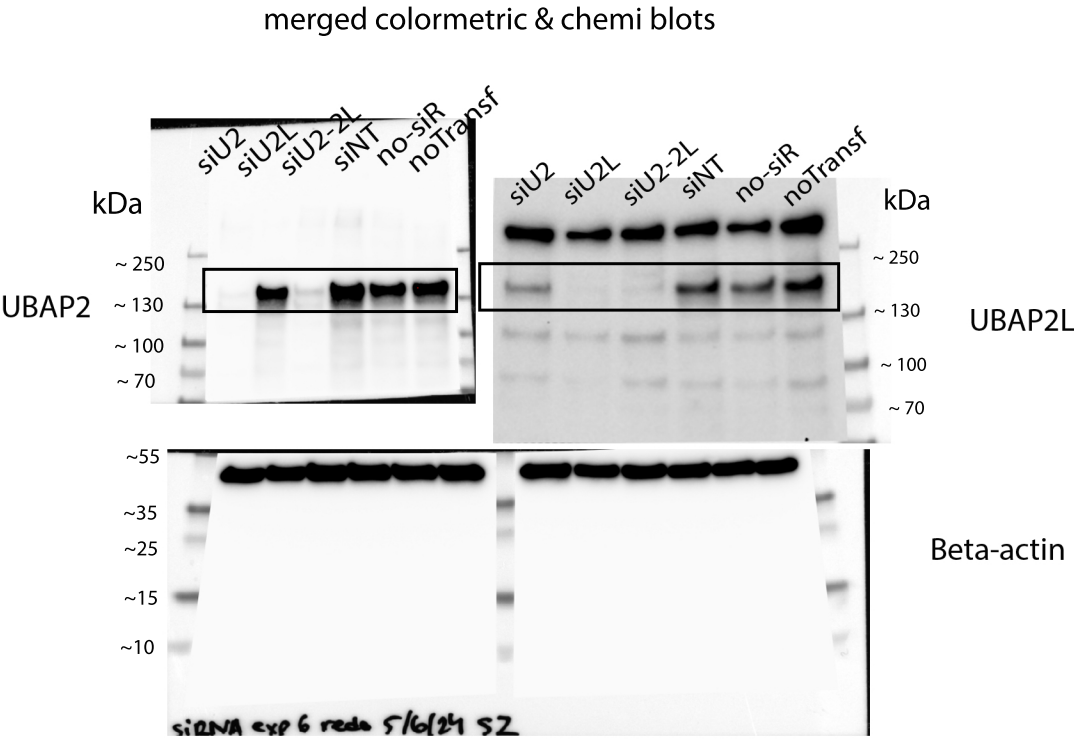

Supplement: SourceData FS1 — is the source file for Fig. S1. [file JCB_202307146_SourceDataFS1.pdf]

colorimetric

chemiluminescence

merged colorm & chemi.

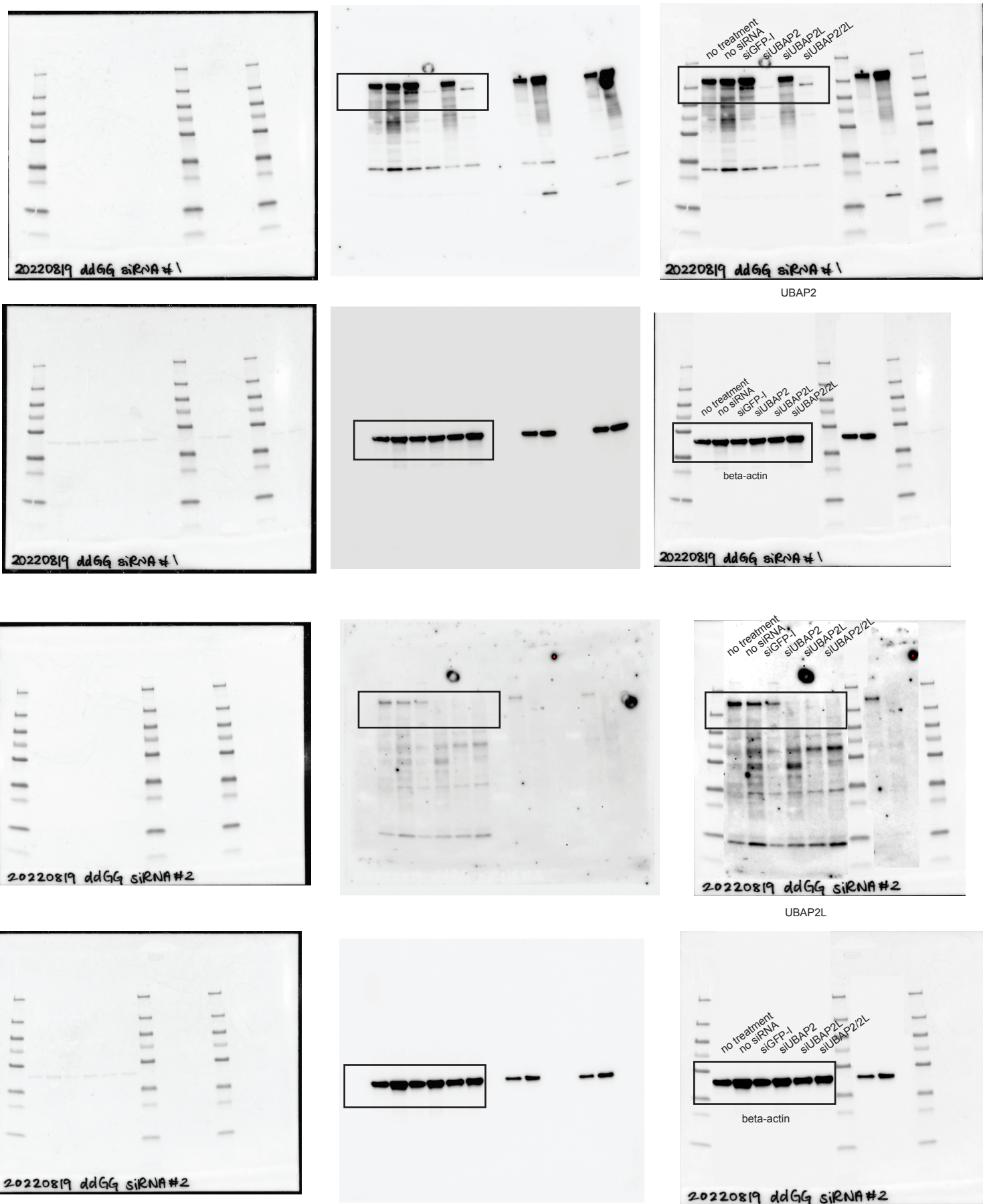

Supplement: SourceData FS2 — is the source file for Fig. S2. [file JCB_202307146_SourceDataFS2.pdf]
